# Ultrasonographic and Histological Correlation after Experimental Reconstruction of a Volumetric Muscle Loss Injury with Adipose Tissue

**DOI:** 10.3390/ijms22136689

**Published:** 2021-06-22

**Authors:** Fernando Leiva-Cepas, Alberto Benito-Ysamat, Ignacio Jimena, Fernando Jimenez-Diaz, Maria Jesus Gil-Belmonte, Ignacio Ruz-Caracuel, Rafael Villalba, Jose Peña-Amaro

**Affiliations:** 1 Research Group in Muscle Regeneration, Department of Morphological Sciences, Faculty of Medicine and Nursing, University of Cordoba, 14004 Cordoba, Spain; fleivacepas@gmail.com (F.L.-C.); benitoysamat@gmail.com (A.B.-Y.); cm1jimei@uco.es (I.J.); mjgilbelmonte@gmail.com (M.J.G.-B.); iruzcaracuel@gmail.com (I.R.-C.); 2 Department of Pathology, Reina Sofia University Hospital, 14004 Cordoba, Spain; 3 Maimonides Institute for Biomedical Research IMIBIC, Reina Sofia University Hospital, University of Cordoba, 14004 Cordoba, Spain; 4 Radiology Department, Musculoskeletal Section, Reina Sofia University Hospital, 14004 Cordoba, Spain; 5 Sport Sciences Faculty, Castilla La Mancha University, 45071 Toledo, Spain; docjimenez58@gmail.com; 6 Department of Health Sciences, Faculty of Medicine, Campus de los Jerónimos, San Antonio Catholic University (UCAM), 30107 Murcia, Spain; 7 Department of Pathology, Ramon y Cajal University Hospital, IRYCIS, 28034 Madrid, Spain; 8 Tissue of Establishment of the Center for Transfusion, Tissues and Cells, 14004 Cordoba, Spain; rafael.villalba.sspa@juntadeandalucia.es

**Keywords:** muscle ultrasound, muscle regeneration, volumetric muscle loss, adipose tissue, muscle tissue engineering

## Abstract

Different types of scaffolds are used to reconstruct muscle volume loss injuries. In this experimental study, we correlated ultrasound observations with histological findings in a muscle volume loss injury reconstructed with autologous adipose tissue. The outcome is compared with decellularized and porous matrix implants. Autologous adipose tissue, decellularized matrix, and a porous collagen matrix were implanted in volumetric muscle loss (VML) injuries generated on the anterior tibial muscles of Wistar rats. Sixty days after implantation, ultrasound findings were compared with histological and histomorphometric analysis. The muscles with an autologous adipose tissue implant exhibited an ultrasound pattern that was quite similar to that of the regenerative control muscles. From a histological point of view, the defects had been occupied by newly formed muscle tissue with certain structural abnormalities that would explain the differences between the ultrasound patterns of the normal control muscles and the regenerated ones. While the decellularized muscle matrix implant resulted in fibrosis and an inflammatory response, the porous collagen matrix implant was replaced by regenerative muscle fibers with neurogenic atrophy and fibrosis. In both cases, the ultrasound images reflected echogenic, echotextural, and vascular changes compatible with the histological findings of failed muscle regeneration. The ultrasound analysis confirmed the histological findings observed in the VML injuries reconstructed by autologous adipose tissue implantation. Ultrasound can be a useful tool for evaluating the structure of muscles reconstructed through tissue engineering.

## 1. Introduction

Ultrasound is an extremely important tool in the assessment of both normal and pathological skeletal muscle structure. Ultrasound studies are mainly focused on the diagnosis and follow-up of neuromuscular diseases [1,2,3,4] and traumatic injuries [5,6]. In these cases, the fundamental aim of the aforementioned studies is to analyze changes in muscle histoarchitecture caused by degenerative and regenerative processes, changes in the size of the muscle fibers, formation of edema, replacement by fibrous and/or adipose tissue, changes in vascularization, etc., all of which translate into changes in ultrasound patterns [7].

One of the situations in which the histoarchitecture of skeletal muscle can be most altered is following muscle reconstruction procedures with tissue engineering techniques. Volumetric muscle loss (VML) injury is the experimental model most commonly used in this type of studies [8,9,10]. In these models, different types of scaffolds, with or without cells, are implanted in the injury area. In conjunction with the regenerative capacity of the host muscle itself, these materials may or may not promote the formation of new muscle tissue [11,12,13]. In this situation, it is important not only to achieve functional recovery, but also to determine whether a structure like that of healthy muscle is generated. To address this matter, ultrasound analysis is regarded as a very useful tool in combination with histological studies. However, few studies on skeletal muscle tissue engineering incorporate ultrasound analyses to correlate their findings with histological ones. The integration of bioscaffolds or materials into skeletal muscle has been followed by ultrasound assessment in a human trial [14] and an equine model [15], with the ultrasound analyses primarily being used to locate the implants in both studies. This tool has also been used to conduct guided biopsies of scaffold implantation territories with a view to assess the characteristics of the newly formed muscle [16].

Ultrasound patterns can be correlated with microscopic changes occurring in cases of muscle atrophy, fibrosis, vascular changes, as well as muscle fiber degeneration and regeneration processes [17,18,19,20]. These changes occur, to a greater or lesser extent, when different materials are used to reconstruct VML injuries [11,13,16,21,22,23]. As in the case of neuromuscular disease, where histological changes in the muscle alter the ultrasound images [7,20], the structural characteristics of tissue engineered muscles can generate specific ultrasound patterns. In a recent study, we experimentally proved that the implantation of autologous adipose tissue in a VML injury significantly favored muscle neoformation, albeit with structural abnormalities [24]. The purpose of this study is to qualitatively correlate the histological and ultrasonographic features of anterior tibial muscles of rats with a VML injury 60 days after undergoing an autologous adipose tissue implantation, upon completion of the regenerative process. The histological and ultrasound patterns were compared with those observed after the implantation of a decellularized muscle matrix or a porous collagen matrix, as well as with those of standard regeneration controls.

## 2. Results

From a histological point of view, in the regenerative control (RC) and fibrotic control (FC) groups, the injuries evolved, as expected, toward complete regeneration and fibrosis, respectively. The evolution of the adipose tissue implants and the two scaffolds used to reconstruct the defects caused by the VML injuries differed with regard to the characteristics of the newly formed muscle tissue. Table 1 summarizes the ultrasound findings, whereas Table 2 summarizes the histomorphometric characteristics of the newly formed muscle in the different study groups.

**Figure 1:**
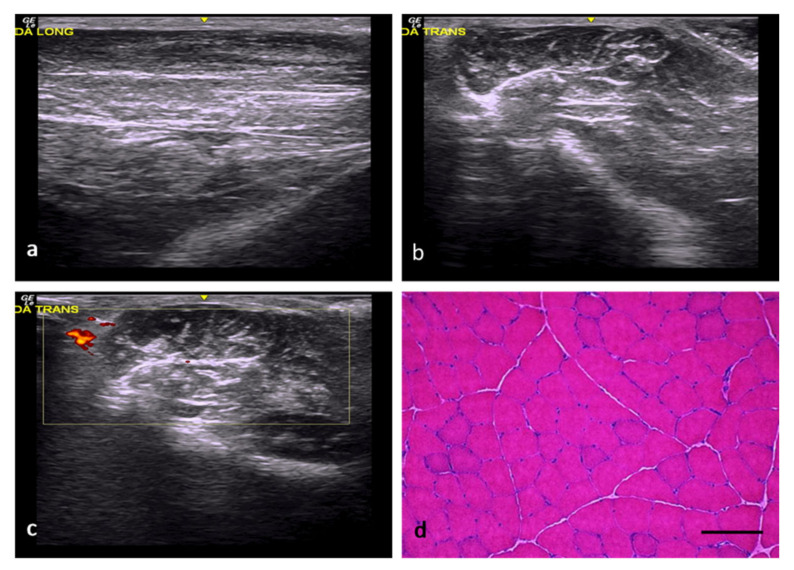
NC group. (**a**) Normal muscle echostructure along the longitudinal axis, comprising a hypoechoic background with numerous hyperechoic lines corresponding to the perimysial septa. (**b**) Normal muscle echostructure along the transverse axis, with a predominantly hypoechoic background with small, interspersed hyperechoic lines and dots corresponding to the perimysium. (**c**) Transverse section assessed by power Doppler imaging, with images of scarce vascularization in the surrounding connective tissue. (**d**) Transverse section representative of the muscle histology. H-E. Size scale: 100 µm.

In the rats of the normal control (NC) group, the muscles exhibited a normal echogenicity and echostructure formed by hypoechoic muscle fibers interspersed with fibrous septa and echogenic fasciae (Figure 1a–c). Along the longitudinal axis, the normal echostructure of the muscle comprised a background of relatively hypoechoic bands corresponding to the muscle fibers interspersed with numerous linear hyperechoic bands corresponding to the fibroadipose septa (perimysium). The same hypoechoic background (muscle fibers) was also observed along the transverse axis, in addition to countless interspersed and disorganized dots and small hyperechoic lines (perimysium) that generated a “starry sky” appearance. The epimysium was identified along both axes, together with a well-defined hyperechoic linear band enveloping the predominantly hypoechoic muscle. Doppler imaging under normal conditions allowed the identification of small, isolated vessels in the connective tissue surrounding the muscle fibers. From a histological point of view, these muscle fibers had rounded or polygonal profiles, peripheral nuclei, and were grouped in bundles (Figure 1d).

**Figure 2:**
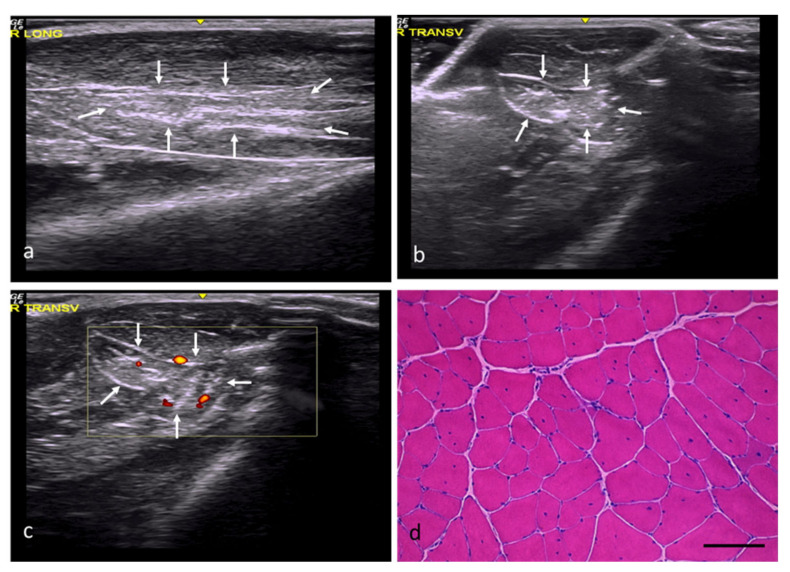
RC group. (**a**) Longitudinal section, with images of a slightly hyperechoic injury compared with the adjacent muscle tissue and a distorted fibrillar pattern, demarcated by white arrows. (**b**) Transverse section, with images of an injury with slightly increased echogenicity and poorly defined margins, demarcated by white arrows. (**c**) Transverse section assessed by power Doppler imaging, with images of moderately increased perilesional vascularization. (**d**) Transverse section representative of the muscle histology. H-E. Size scale: 100 µm.

In the RC group, the injury areas could be identified, but their margins were imprecise and poorly defined, with a slightly distorted echostructure, somewhat increased echogenicity, and moderately increased vascularization (Figure 2a–c). In this study group, the muscles’ histological features were practically normal, except for internal nuclei (Figure 2d) and some minimal changes (Table 2).

**Figure 3:**
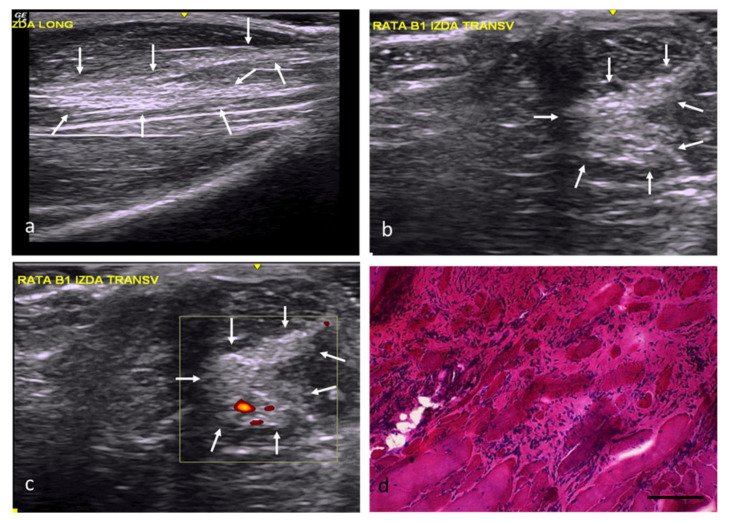
FC group. (**a**) Longitudinal axis, with images of a hyperechoic area compared with the normal muscle tissue and a distorted echostructure, demarcated by white arrows. (**b**) Transverse axis, with images of a well-defined injury with angular margins and increased echogenicity. (**c**) Transverse section assessed by power Doppler imaging, with images of a slight, predominantly peripheral, increase in vascularization. (**d**) Transverse section representative of the muscle histology. H-E. Size scale: 100 µm.

In the FC group, the injuries had an angular morphology, were well defined, and had slightly increased echogenicity, a clearly distorted echostructure, and slightly increased vascularization (Figure 3a–c). From a histological point of view, 74% of the area of the muscle defects in this group was occupied by fibroadipose tissue (Figure 3d). The scarce number newly formed fibers, some with internal nuclei, were relegated to the injuries’ margins (Figure 3d).

**Figure 4:**
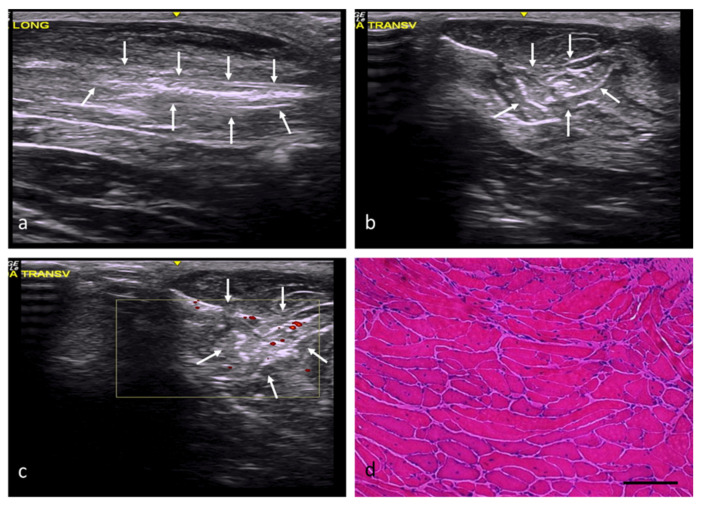
AT group. (**a**) Longitudinal axis, with images of a hyperechoic injury with poorly defined margins, an imprecise morphology, and a slightly distorted echostructure, demarcated by white arrows. (**b**) Transverse axis, with images of an injury with slightly increased echogenicity, poorly defined margins, and an imprecise morphology. (**c**) Transverse section assessed by power Doppler imaging, with images of slightly increased intralesional vascularization. (**d**) Transverse section representative of the muscle histology. H-E. Size scale: 100 µm.

In the adipose tissue (AT) group, the injury margins were slightly unclear, although the implantation areas were hyperechogenic and had a slightly distorted echostructure (Figure 4a–c). According to the histological study, the adipose tissue implanted in the defects had been mostly replaced by skeletal muscle tissue (Figure 4d), with a percentage of fibrosis of approximately 20%. The newly formed muscles exhibited variations in terms of the orientation and size of their fibers, with some being large and others clearly atrophic, but all of them with central or internal nuclei (Table 2).

**Figure 5:**
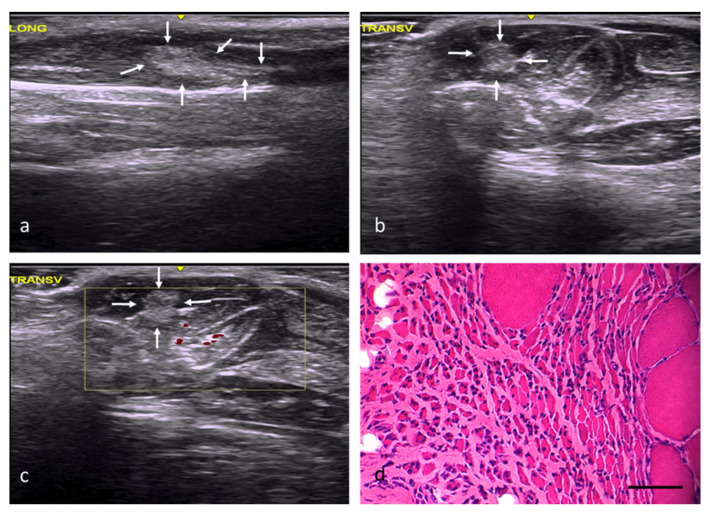
OS group. (**a**) Longitudinal axis, with images of a well-defined injury with increased echogenicity and a clearly distorted echostructure compared with the adjacent tissue. (**b**) Transverse axis, with images of an oval injury with well-defined margins. (**c**) Transverse section assessed by power Doppler imaging, with images of slightly increased perilesional vascularization. (**d**) Transverse section representative of the muscle histology. H-E. Size scale: 100 µm.

**Figure 6:**
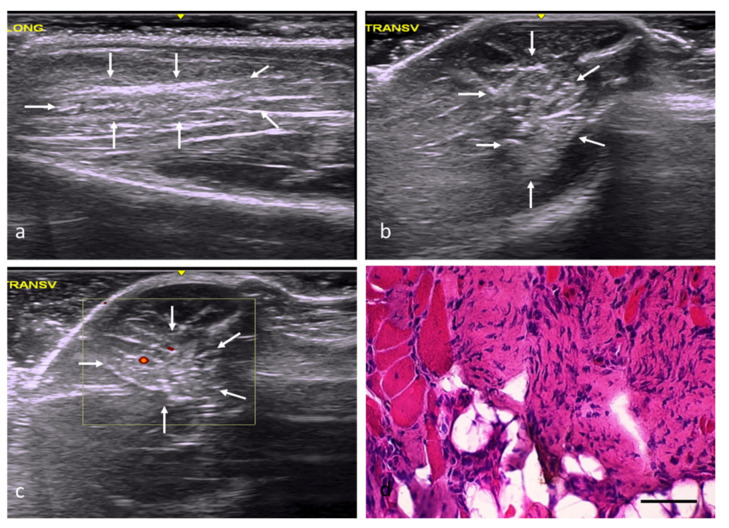
DM group. (**a**) Longitudinal axis, with images of an injury with increased echogenicity and a distorted echostructure compared with the adjacent muscle tissue. (**b**) Transverse axis, with images of a well-defined, oval injury with increased echogenicity. (**c**) Transverse section assessed by power Doppler imaging, with images of a slight, predominantly peripheral, increase in intralesional vascularization. (**d**) Transverse section representative of the muscle histology. H-E. Size scale: 100 µm.

In the Osteovit^®^ (OS) and heterologous decellularized muscle matrix (DM) groups, the injury margins were well defined, and the injury area had a variable morphology. In both cases, the echogenicity was increased and the echotexture severely distorted (Figure 5a–c, Figure 6a–c). In the OS group, the percentage of fibrosis reached approximately 31%. However, although the implantation of the scaffold in this group did not prevent the generation of new muscle fibers, most of these fibers exhibited atrophic histological and histomorphometric features secondary to denervation (Figure 5d). The connective tissues’ thickness contained complete bundles of atrophic fibers, some of which were found to coexist with very large fibers (suggestive of reinnervation). The muscle fibers reached maturity in certain areas where reinnervation of the regenerative muscle fibers was not impeded, albeit with some morphological abnormalities with regard to their shape and size.

In the DM group, the decellularized matrix implantations resulted in significant fibrosis (68%), with a very scarce number of newly formed, small, and overall disoriented fibers (Figure 6d) (Table 2). In addition, foci of a chronic inflammatory infiltrate with multinucleated giant cells were found among of the fibroadipose tissue.

**Figure 7:**
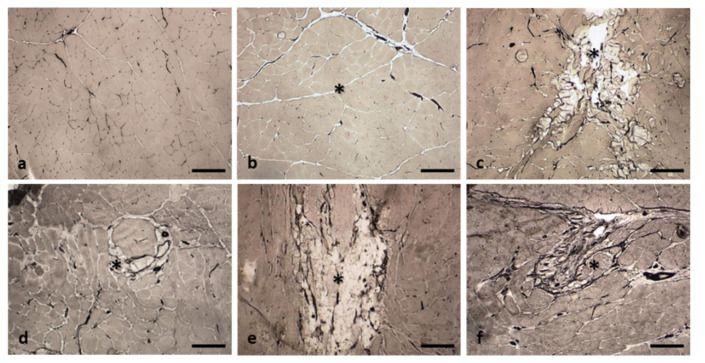
Representative images of the central area of injury in each group. The vascular network is stained black with the ATPase histochemical technique. In the NC group, the vascular network is poorly marked (**a**). In the RC (**b**) and OS (**e**) groups, greater vascularization is observed in the periphery of the regenerated and implantation areas, respectively (asterisks). In the FC (**c**), AT (**d**) and DM (**f**) groups, vascularization is more increased within the implantation zone (asterisks). ATPase pH 4.2. Size scale: 100 µm.

A color Doppler ultrasound revealed moderately increased vascularization in the injuries of all groups, although this increase was perilesional in the RC and OS groups and intralesional in the FC, AT, and DM groups (Figure 2, Figure 3, Figure 4, Figure 5 and Figure 6). Moreover, through ATPase staining, it was possible to identify the vascular network and confirm the increased distribution (Figure 7) observed in the ultrasound analysis.

## 3. Discussion

Ultrasounds detect changes in the histoarchitecture of skeletal muscle, which are seen as changes in the echotexture and echogenicity, thus enabling the diagnosis and follow-up of neuromuscular diseases [1,2,3], traumatic injuries [5,25,26], and experimental models of muscle disorders [16,17,18,19,27,28].

In this study, we correlated ultrasound observations with histological findings of the anterior tibial muscles of rats 60 days after implanting different scaffolds in a muscle defect caused by a VML injury. The ultrasound analysis showed that the muscles of the rats of the AT group tended to exhibit normal ultrasonographic features that were similar to those of the rats of the RC group. In contrast, those of the rats of the FC, OS, and MD groups were characterized by a clearly altered image, albeit with no evident differences in the ultrasound patterns of the different groups. These ultrasound findings correlated well with the histological observations.

Recognizing the margins and morphology of VML injuries can be useful in determining the degree of tissue reconstruction. These margins can be identified based on the contrast between the echotexture and echogenicity of the normal tissue and that of the injury. In our study, the skeletal muscles of the RC group’s rats were found to have a greater tendency to exhibit normal ultrasonographic and histological features (the latter with the characteristic presence of internal nuclei within the muscle fibers). This finding is consistent with that reported in a previous study, in which anterior tibial muscles were fully regenerated and had a normal ultrasound pattern merely 30 days after the injection of mepivacaine [19]. This would explain why the injury margins in the RC group were not discernible and their morphology imprecise. Thus, the fact that the injuries in the AT group were poorly delimited and had an undefined morphology suggests that the defects had been occupied by newly formed muscle tissue. On the contrary, the clear demarcation of the injuries and their recognizable, but variable, morphology in the FC, OS, and DM groups was indicative of failed muscle reconstruction.

In skeletal muscle degeneration–regeneration, the degeneration phase is characterized by increased echogenicity. In contrast, the regeneration phase is characterized by progressively decreasing hyperechogenicity and recovery of a homogeneous texture until the attainment of normality [1,19,27]. In the AT, OS, and MD implantation groups, the injured areas remained hyperechogenic, thus suggesting that they did not regain histological normality. However, the echostructure of the injuries did differ between the three groups. For example, the echotexture of the injuries in the AT group was similar to that of those in the RC group (in which normal regeneration had been achieved), with 80% of the area of the volumetric defects being occupied by newly formed muscle tissue, albeit with significant structural abnormalities in terms of the varying size and spatial disorientation of the new muscle fibers. The increased echogenicity would be explained by this disorganization of the muscle histoarchitecture.

Although increased echogenicity is indicative of a more disorganized muscle histoarchitecture, infiltration by adipose tissue and the appearance of edema can also contribute to this finding [28,29]. The FC and DM groups were histologically characterized by a 75% and 68% replacement of the VML injury area by fibroadipose tissue, respectively, although regenerative muscle fibers had also formed along the injuries’ margins. When this regeneration is impeded, fibroadipogenic progenitors, and even the satellite cells themselves [30,31], migrate from the surviving area to promote repair with fibroadipose tissue. Therefore, this combination of tissues in muscle defects must be responsible for the heterogeneous echotexture observed among these groups.

The most characteristic histological feature in the OS group was the presence of extensive atrophy of fascicular distribution, which clearly indicates the existence of innervation failures in the newly formed muscle fibers. Compared with the FC group, where fibrosis reached 74%, only 31% fibrosis was observed in the OS group. Although the collagen scaffold did not inhibit the regenerative response of the skeletal muscle, it did appear to hinder the growth of new muscle fibers by favoring the formation of fibrotic bands and newly formed tendons (unpublished observations). This correlates well with an ultrasound pattern characterized by a loss of the hypoechoic pattern of the muscle fibers and increased hyperechogenicity. In muscle atrophy secondary to denervation, the presence of atrophic fibers correlates with homogeneous structures of increased echogenicity [32] due to the hypoechoic loss of muscle fibers and the growth of connective tissue.

Hyperechogenicity is also the usual pattern described in muscle lesions occupied by degenerating muscle fibers and inflammatory infiltrate [17,19,33]. Coinciding with the findings reported by other authors [34,35], in our study, the decellularized matrix implantation did not favor the regenerative response of the host muscle to reconstruct the muscle defect. The histological pattern observed in the DM group was characterized by the presence of an inflammatory response and fibroadipose tissue, which also correlate well with the increased echogenicity and heterogeneous echotexture findings.

Revascularization is an essential process in muscle fiber regeneration [36]. Under normal conditions, color Doppler imaging reveals scarce vascularization and, once the regeneration process of the injury is complete, no images of revascularization are identified [19]. Conversely, during early stages of regeneration, vascularization is abundant and easily viewed accompanying the myotubes or regenerative muscle fibers with color Doppler imaging [19]. In this study, the presence of moderately increased vascularization, both perilesional and intralesional, in the ultrasound analysis, which was confirmed by ATPase histochemical staining, could be indicative of different aspects. In the case of the AT group, the presence of an intralesional increase in vascularization could be indicative of significant vascularization associated with the newly formed muscle fibers, possibly favored by the proangiogenic activity of the stromal cells of the adipose tissue [37]. However, this ultrasound finding can also be detected in inflammatory conditions [7], which would explain the intralesional increase in the DM group. In the case of the OS group, the limited vascularization in the perilesional area of the injuries could be attributed to the decrease in the number of vessels accompanying the atrophic muscle fibers [38].

One limitation of this study was the lack of ultrasound follow-up of the evolution of the injuries following the implantations. Specifically, this follow-up was not carried out during the early post-implantation phases because the surgical wound prevented the ultrasound study from being conducted. Although it could be interesting to determine the ultrasound stages of regeneration/repair during muscle reconstruction in this model, we believe that it might be more useful to determine the histoarchitecture of the newly formed muscle upon completion of the process.

In conclusion, the ultrasound analysis confirmed the histological findings observed in the VML injuries reconstructed by autologous adipose tissue implantation. Although the ultrasound pattern was found to differ from that observed in the fully regenerated injuries of the RC group, it was clearly different from that of the groups (OS and DM) in which the generation of new muscle had failed. Through these observations, it is possible to define ultrasonographic and histological reference standards to aid in assessing the integrity of the regenerative response in skeletal muscle tissue engineering using non-invasive and less expensive techniques.

In our opinion, besides its usefulness as an aid in both the diagnosis of neuromuscular disorders and the follow-up of muscle injuries, ultrasound can also serve as a useful tool for evaluating the structure of new muscles formed by tissue engineering.

## 4. Materials and Methods

### 4.1. Animals

A total of 24 male Wistar rats, weighing 350 ± 50 g, were used in this study. The animals were divided into different groups and housed in cages with access to water and food ad libitum, a constant temperature of 22 °C, and 12 h cycles of light/darkness. All procedures were approved by the Ethics Committee on Animal Experimentation of the University of Cordoba (ethical approval code 2017PI/05) (Spain) and of the Regional Government of Andalusia (ethical approval code 07/09/2017/121) (Spain).

### 4.2. Volumetric Muscle Loss Injuries and Animal Groups

All procedures (surgery, ultrasound diagnosis, and sacrifice) were performed under general anesthesia (ketamine/medetomidine at a dose of 7.5/0.05 mg/100 g of body weight) and aseptic conditions (cutaneous chlorhexidine), where appropriate.

The animals were divided into three experimental groups and three controls (4 rats each). The experimental groups, as well as the negative control group, were formed by rats on which a VML injury had been generated on the central portion of their anterior tibial muscle by punch extraction of a cylindrical tissue fragment (6 mm in diameter and 5 mm in length).

**Figure 8:**
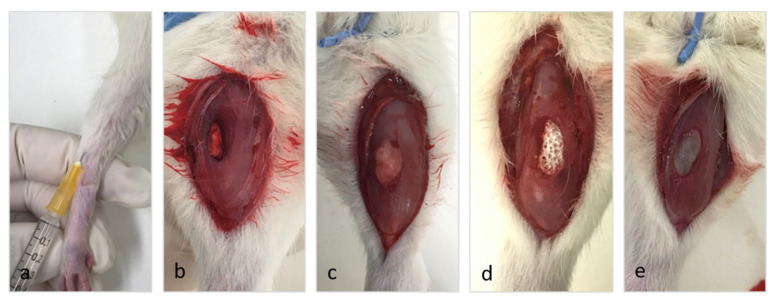
Control and experimental groups. (**a**) Intramuscular injection of mepivacaine (RC group). Implantation in the muscle defect of: (**b**) A resorbable hemostatic sponge (FC group), (**c**) autologous adipose tissue (AT group), (**d**) Osteovit^®^ (OS group), or (**e**) a decellularized muscle matrix (DM group).

The control groups comprised the normal control group (NC group), the regenerative control (RC group) or positive control group, and the fibrotic control (FC group) or negative control group. The RC group included rats that had received an intramuscular injection of mepivacaine, a local anesthetic (Scandinibsa^®^, Inibsa, Barselona, Spain) (Figure 8a). Given that VML injuries evolve toward complete regeneration in this type of group, it is typically used as a comparative reference of a regular and complete regenerative process [9,39]. The FC group, or negative control, included rats in which a muscle fragment was excised without the subsequent implantation of any material, except for a hemostatic sponge (Gellita-Spon^®^ standard, Gellita Medical GmbH, Eberbach, Germany) to prevent bleeding (Figure 8b). In this type of group, volumetric defects evolve into a fibrous scar [8]. The animals of the NC group were not subjected to any sort of experimental procedure.

The animals of the experimental groups had autologous adipose tissue (AT group), Osteovit^®^ (OS group), or a heterologous decellularized muscle matrix (DM group) implanted in their VML injuries (Figure 8c–e). In the AT group, autologous adipose tissue sourced from the inguinal region of each subject was implanted in the injury as previously described [24]; briefly, in the same surgical act, a fragment of adipose tissue from the inguinal region of the same animal was implanted in the volumetric defect, equivalent to the extracted muscle fragment of approximately 0.07 g. Meanwhile, a commercial porous collagen matrix (Osteovit^®^, B. Braun, Melsungen, Spain) was implanted in the OS group; this was used in our study because collagen is the main structural protein in the extracellular matrix of skeletal [40] and is an important element in the preparation of scaffolds for muscle tissue engineering [41]. In the DM group, heterologous decellularized muscle matrices sourced from the soleus muscles of four normal rats following a standardized procedure were used [42] with some modifications; briefly, after removing the tendon ends and fasciae, the muscles were successively kept in agitation at room temperature in: 1% sodium dodecyl sulfate (SDS) for 24 h, in PBS for 24 h and Triton X-100 solution, 0.5% for 24 h. Subsequently, they were treated with a DNase solution for 3 h at 37 °C and then left the tissue in PBS and stored at 4 °C.

### 4.3. Skeletal Muscle Ultrasound Evaluation

The rats were examined with a muscle ultrasound performed under general anesthesia 60 days after the implantation procedure. After administering the anesthesia, the animals were placed in a prone position, the ultrasound examination area was shaved, and an antiseptic (chlorhexidine) was applied on the area before performing the ultrasound analysis [19].

A General Electric LOGIQ e BT12 portable ultrasound machine (General Electrics, Boston, MA, USA) with a high-frequency linear probe (8–18 MHz) was used for this analysis. The anterior region of each subject’s hind limb was evaluated bilaterally, obtaining images in the transverse and longitudinal planes in B mode, as well as with power Doppler imaging, adjusting the focus on the area of interest. In B mode, the described region was assessed with the following settings: Tissue harmonic imaging (THI) frequency 16 MHz, gain 32, enhancement 3, average 2, and dynamic range 96 dB. The same region was subsequently also evaluated by power Doppler imaging with the following settings: Frequency 10 MHz, gain of 12, pulse repetition frequency 0.8 kHz, and wall filter 89 Hz.

Ultrasound images were classified using the following classification system to assess increased echogenicity, echotexture distortion, and vascularity: Grade 0 (normal), grade 1 (mild), grade 2 (moderate), and grade 3 (severe/high). The ultrasound analysis was performed by a blinded, experienced, and specialist radiologist (ABY).

### 4.4. Histology and Histomorphometry

Sixty days after the implantation, the rats were sacrificed under anesthesia and their anterior tibial muscles were excised, oriented for cross-sections, embedded in OCT Compound, and flash-frozen in isopentane cooled (−160 °C) in liquid nitrogen. A cryostat (Leica CM1850 UV, Leica Microsystems, Nussloch, Germany) at −20 °C was used to obtain 8 µm-thick sections of the muscles. These sections were subsequently stained with hematoxylin and eosin (H-E) for their morphological analysis and with adenosine triphosphatase (ATPase, pH 4.2) histochemical stain for vascular identification [43,44].

Five areas located within the implantation site of each muscle were photographed with a 40× objective using a Nikon Eclipse E1000 microscope (Nikon, Tokyo, Japan) incorporating a Sony DXC-990P color video camera (Sony, Tokyo, Japan). The images were then transferred to a computer equipped with the Image-ProPlus 6.5 image analysis software (Media Cybernetics, Bethesda, MA, USA). The following parameters indicative of the degrees of regeneration and fibrosis were evaluated in each of these areas: Cross-sectional area of the muscle fibers (µm2), minor diameter of the muscle fibers (µm), total area of the image covered by connective tissue (µm2), number of muscle fibers/area, number of muscle fibers with internalized nuclei/area, and number of disoriented (non-transverse) fibers/area. Some of the histomorphometric results of the NC, CR and FC groups in Table 2 have been previously published by our group. This has been done for ethical reasons in order to reduce the number of test animal [24].

### 4.5. Statistical Analysis

The statistical analysis was carried out using statistical software SigmaStat 3.1 (Systat Software Inc., Point Richmond, CA, USA). To perform such analysis, the mean of the five areas was calculated for each sample, following which the mean ± standard deviation was determined for each group. Statistical significance (*p* < 0.05) between groups was determined by ANOVA on ranks. In those samples that met normality criteria, the Holm–Sidak *t*-test was performed, and in the rest, the Dunn test was used.

## Figures and Tables

**Table 1:** Summary of the ultrasound findings in the implantation area of the VML lesion with the tissue/biomaterial used after 60 days of evolution.

Group	Identification of the Lesion	Edges of Lesion	Morphology of the Lesion	Increased Echogenicity	Echotexture Distortion	Vascularization
NC	—	—	—	Grade 0	Grade 0	Grade 0
RC	Yes	poorly delimited	imprecise	Grade 1	Grade 1	Perilesional Grade 2
FC	Yes	well delimited	angulated	Grade 3	Grade 3	Intralesional Grade 2
AT	Yes	poorly delimited	imprecise	Grade 2	Grade 1	Intralesional Grade 1
OS	Yes	well delimited	Variable (rounded, angled, or patchy)	Grade 3	Grade 3	Perilesional Grade 1
DM	Yes	well delimited	Variable (rounded, angled, or patchy)	Grade 2	Grade 3	Intralesional Grade 1

NC: normal control; RC: regenerative control; FC: fibrotic control; AT: adipose tissue; OS: Osteovit^®^; DM: decellularized matrix. Grade 0 (normal); Grade 1 (mild); Grade 2 (moderate); Grade 3 (severe/high).

**Table 2:** Histomorphometric characteristics of the neomuscle formed within VML injury with the tissue/biomaterial used after 60 days of evolution.

Group	Fibrosis (%)	Number of Muscle Fibers/Area	Cross-Sectional Area of Muscle Fibers (µm^2^)	Minor Diameter of Muscle Fibers (µm)	Muscle Fibers with Internal Nuclei (%)	Desorientated Muscle Fibers (%)
**NC**	2.89 ± 0.25	14.8 ± 0.7	3400.6 ± 184.4	50.4 ± 2.0	4.2 ± 1.63	0.0 ± 0.0
**RC**	5.80 ± 0.52 *	14.8 ± 0.4	3514.8 ± 153.8	49.08 ± 1.8	68.8 ± 3.4 *	0.9 ± 0.1 *
**FC**	74.33 ± 3.49 * †	7.0 ± 0.6 * †	1505.7 ± 207.4 * †	31.4 ± 1.3 * †	39.6 ± 3.3 * †	3.2 ± 0.9 * †
**AT**	19.57 ± 3.47 * † §	16.8 ± 0.7 §	2385.0 ± 241.0 * † §	29.8 ± 1.2 * †	62.3 ± 5.1 * §	23.9 ± 3.0 * † §
**OS**	30.65 ± 2.58 * † § #	71.0 ± 19.0 * † §	536.2 ± 41.0 * † § #	17.4 ± 2.2 * † § #	42.8 ± 4.7 * † #	20.5 ± 4.8 * † §
**DM**	67.7 ± 5.78 * † # ‡	10.1 ± 1.2 * † # ‡	1536.8 ± 159.8 * † # ‡	27.5 ± 1.9 * † ‡	48.7 ± 5.1 * † #	39.2 ± 7.23 * † § # ‡

All values are expressed as mean ± SD. * *p* < 0.05 vs. NC group; † *p* < 0.05 vs. RC group; § *p* < 0.05 vs. FC group; # *p* < 0.05 vs. AT group; ‡ *p* < 0.05 vs. OS group.

## Data Availability

The data presented in this study are available on request from the corresponding author.
